# Human-genome single nucleotide polymorphisms affecting transcription factor binding and their role in pathogenesis

**DOI:** 10.18699/VJGB-23-77

**Published:** 2023-10

**Authors:** E.V. Antontseva, A.O. Degtyareva, E.E . Korbolina, I.S. Damarov, T.I. Merkulova

**Affiliations:** Institute of Cytology and Genetics of the Siberian Branch of the Russian Academy of Sciences, Novosibirsk, Russia; Institute of Cytology and Genetics of the Siberian Branch of the Russian Academy of Sciences, Novosibirsk, Russia; Institute of Cytology and Genetics of the Siberian Branch of the Russian Academy of Sciences, Novosibirsk, Russia; Institute of Cytology and Genetics of the Siberian Branch of the Russian Academy of Sciences, Novosibirsk, Russia; Institute of Cytology and Genetics of the Siberian Branch of the Russian Academy of Sciences, Novosibirsk, Russia

**Keywords:** regulatory single-nucleotide polymorphism, transcription factor-binding sites, gene expression, genome-wide studies, регуляторный однонуклеотидный полиморфизм, сайты связывания транскрипционных факторов, экспрессия генов, полногеномные исследования

## Abstract

Single nucleotide polymorphisms (SNPs) are the most common type of variation in the human genome. The vast majority of SNPs identified in the human genome do not have any effect on the phenotype; however, some can lead to changes in the function of a gene or the level of its expression. Most SNPs associated with certain traits or pathologies are mapped to regulatory regions of the genome and affect gene expression by changing transcription factor binding sites. In recent decades, substantial effort has been invested in searching for such regulatory SNPs (rSNPs) and understanding the mechanisms by which they lead to phenotypic differences, primarily to individual differences in susceptibility to diseases and in sensitivity to drugs. The development of the NGS (next-generation sequencing) technology has contributed not only to the identification of a huge number of SNPs and to the search for their association (genome-wide association studies, GWASs) with certain diseases or phenotypic manifestations, but also to the development of more productive approaches to their functional annotation. It should be noted that the presence of an association does not allow one to identify a functional, truly disease-associated DNA sequence variant among multiple marker SNPs that are detected due to linkage disequilibrium. Moreover, determination of associations of genetic variants with a disease does not provide information about the functionality of these variants, which is necessary to elucidate the molecular mechanisms of the development of pathology and to design effective methods for its treatment and prevention. In this regard, the functional analysis of SNPs annotated in the GWAS catalog, both at the genome-wide level and at the level of individual SNPs, became especially relevant in recent years. A genome-wide search for potential rSNPs is possible without any prior knowledge of their association with a trait. Thus, mapping expression quantitative trait loci (eQTLs) makes it possible to identify an SNP for which – among transcriptomes of homozygotes and heterozygotes for its various alleles – there are differences in the expression level of certain genes, which can be located at various distances from the SNP. To predict rSNPs, approaches based on searches for allele-specific events in RNA-seq, ChIP-seq, DNase-seq, ATAC-seq, MPRA, and other data are also used. Nonetheless, for a more complete functional annotation of such rSNPs, it is necessary to establish their association with a trait, in particular, with a predisposition to a certain pathology or sensitivity to drugs. Thus, approaches to finding SNPs important for the development of a trait can be categorized into two groups: (1) starting from data on an association of SNPs with a certain trait, (2) starting from the determination of allele-specific changes at the molecular level (in a transcriptome or regulome). Only comprehensive use of strategically different approaches can considerably enrich our knowledge about the role of genetic determinants in the molecular mechanisms of trait formation, including predisposition to multifactorial diseases.

## Abstract (ru)

Однонуклеотидные замены, также называемые однонуклеотидными полиморфизмами (single nucleotide polymorphism, SNP), – это наиболее распространенный тип вариаций генома человека. Подавляющая часть выявленных в геноме человека SNP не оказывает какого-либо воздействия на молекулярный фенотип, однако некоторые способны приводить к изменению функции гена или уровня его экспрессии. В то же время большинство SNP, ассоциированных с некими признаками или патологиями, картируются в регуляторных областях генома, изменяют потенциальные сайты связывания транскрипционных факторов и, соответственно, могут влияют на экспрессию генов. В последние десятилетия значительные усилия были направлены на поиск таких регуляторных SNP (rSNP), а так же на понимание механизмов, посредством которых они приводят к фенотипическим различиям, в первую очередь к разной предрасположенности к заболеваниям и индивидуальной чувствительности к лекарственным препаратам. Развитие технологии NGS (next generation sequencing) способствовало не только выявлению огромного количества SNP и поиску их ассоциации (genome wide association studies, GWAS) с некими заболеваниями или фенотипическими проявлениями, но и развитию более производительных подходов для их функциональной аннотации. Стоит отметить, что наличие ассоциации не позволяет выделить функциональный, действительно связанный с болезнью вариант последовательности ДНК из множества маркерных, которые выявляются за счет неравновесия по сцеплению. Более того, установление ассоциаций генетических вариантов с заболеванием не дает сведений о функциональности этих вариантов, что необходимо для выяснения молекулярных механизмов развития патологии и разработки эффективных методов ее лечения и профилактики. В связи с этим функциональный анализ SNP, аннотированных в GWAS каталоге, как на полногеномном уровне, так и на уровне отдельных SNP в последние годы стал особенно актуальным. В настоящее время активно развивается полногеномный поиск потенциально регуляторных SNP без каких-либо предварительных знаний об их ассоциации с признаком. Так, картирование локусов количественных признаков экспрессии (eQTL, expression quantitative trait loci) позволяет выявить SNP, для которого в транскриптомах гомозигот по разным его аллелям, а также гетерозигот наблюдаются различия в уровне экспрессии неких генов, причем как близко расположенных, так и на значительном удалении. Для предсказания регуляторных SNP используют также подходы, основанные на поиске аллель-специфических событий в данных RNA-seq, ChIP-seq, DNase-seq, ATAC- seq, MPRA и т. д. Однако для более полной характеристики таких rSNP необходимо устанавливать их ассоциацию с признаком, в частности с предрасположенностью к некой патологии или с чувствительностью к лекарственным препаратам. Таким образом, именно комплексное использование двух основанных на противоположных принципах подходов к поиску значимых для развития признака (патологии) SNP: с одной стороны, исходящего из данных по ассоциации SNP с неким признаком, а с другой стороны, идущего от определения аллель-специфичных изменений на молекулярном уровне (в транскриптоме или регуломе) – существенно обогащает картину наших знаний о роли генетических детерминант в молекулярных механизмах формирования признаков, включая предрасположенность к многофакторным заболеваниям.

## Introduction

One of the main tasks of human genetics is to clarify the mechanisms by which genome variations lead to phenotypic differences, primarily to individual differences in susceptibility to diseases and in sensitivity to drugs. Single-nucleotide polymorphisms (SNPs) are the most common type of genome variation (Chanock, 2001). Currently, due to the development of next-generation sequencing (NGS) technologies, more than 950 million SNPs of the human genome are registered in database dbSNP (https://www.ncbi.nlm.nih.gov/projects/ SNP/snp_summary.cgi) (Sherry et al., 2001); furthermore, rare SNPs with a frequency (prevalence in the population) of less than 1 % constitute more than 90 % of the total number of SNPs. It seems unlikely that the vast majority of identified variations can be important for the phenotype, but some of them certainly form the genetic basis of phenotypic traits, including predisposition to various diseases. In recent decades, major efforts have been applied to the search for such SNPs.

The most popular approach (which started back in the 1980s) to the identification of trait-related SNPs has been the determination of associations with diseases for SNPs found in candidate genes (Lander, Schork, 1994; Ring, Kroetz, 2002), and in this context, only SNPs affecting the proteincoding part of a gene have been investigated (Cooper, 1998). Somewhat later, there has been some interest in elucidating the functionality of variants located in noncoding regions of genes, i. e., in determining an effect of such variants on a certain molecular phenotype. In particular, it has been shown that such SNPs affect transcription factor-binding sites (TFBSs), thereby leading to changes in the expression of the respective genes (Ludlow et al., 1996; Piedrafita et al., 1996; Knight et al., 1999; Vasiliev et al., 1999). Nonetheless, these few functional studies have remained almost invisible against the backdrop of a huge wave of research aimed at identifying associations.

**Fig. 1.**
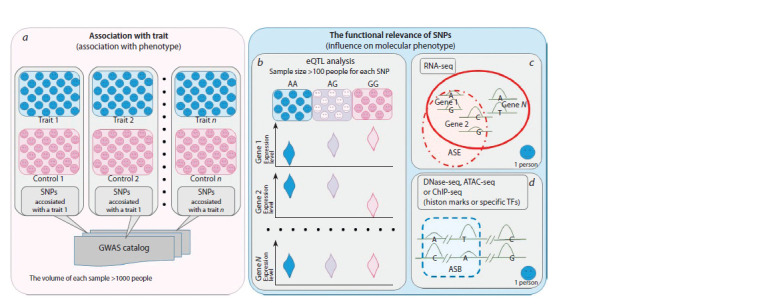
Genome-wide approaches to the analysis of SNPs. a, The principle of GWASs; b, the scheme of eQTL analysis for each SNP. A search for allele-specific events: c, expression (allele-specific expression, ASE) and d, binding (allele-specific binding, ASB).

The productivity of detection of disease-associated SNPs increased dramatically with the advent of the genome-wide association study (GWAS) technology in the mid-2000s (Fig. 1, a), which is based on an unbiased – not based on any ideas about the formation of a trait – principle of a genomewide search for SNPs associated with the trait (Visscher et al., 2012; Tam et al., 2019). To date, more than 72 thousand associations of genetic variants with traits have been found by GWASs (GWAS Catalog, https://www.ebi.ac.uk/gwas/), thus allowing to find many new genes and systems of genes associated with predisposition to various diseases (Buniello et al., 2019; Tam et al., 2019; Claussnitzer et al., 2020).

Nevertheless, GWAS technology does not provide any information about the functionality of the detected variants, thereby making it very difficult to elucidate the molecular mechanisms underlying the development of a pathology and hence to develop effective methods for its treatment and prevention. Additionally, based on results of GWASs, it is almost impossible to distinguish a truly disease-associated variant from the many marker variants that are detected due to linkage disequilibrium (Lappalainen, 2015; Tam et al., 2019; Zhao et al., 2020). It is also known that most SNPs identified by GWASs are located in the noncoding part of the genome and, as a rule, in its regulatory regions (e. g., promoters and enhancers) (Hindorff et al., 2009; Maurano et al., 2012; Bryzgalov et al., 2013; Farh et al., 2015); these data imply an influence of such SNPs on the binding of transcription factors (TFs) and on gene expression. Thus, a need for research on functional interpretation of data from GWASs – both at the level of individual potentially regulatory SNPs (rSNPs) and at the level of all such variants collectively – has become obvious. In addition, because the GWAS technology greatly underestimates the actual number of associations owing to the required strict thresholds in statistical processing (Tam et al., 2019), investigators have recognized the need to develop GWAS-unrelated large-scale function-based approaches to the search for rSNPs (Westra, Franke, 2014; Maurano et al., 2015; Cavalli et al., 2016b; Korbolina et al., 2018).

timing of emergence (almost simultaneous with the start of the application of the GWAS technology) is analysis of expression quantitative trait loci (eQTLs) (see Fig. 1, b). This analysis, by means of transcriptome data (earlier, microarray data have been employed, then data from high-throughput RNA sequencing [RNA-seq] started to be used), determines for each SNP a difference in the level of expression of individual genes among homozygotes and heterozygotes for different alleles of this SNP (Westra, Franke, 2014; GTEx Consortium, 2020). Somewhat later, techniques were devised for finding allele-specific events both in data from RNA-seq (allele-specific expression [ASE] events) (see Fig. 1, c) (Castel et al., 2020; Fan et al., 2020) and in data from ChIP-seq, DNase-seq, and ATAC-seq (allele-specific binding [ASB] events) (see Fig. 1, d ) (Maurano et al., 2015; Cavalli et al., 2016a, b; Xu et al., 2020; Korbolina et al., 2021). Massive function-based approaches also include massively parallel reporter assay (MPRA), SNP-seq, and SNP-SELEX (Zhang et al., 2018; Lu et al., 2021; Yan et al., 2021).

At the beginning of this review, using the latest studies as examples, we examine rSNPs that are (i) associated with the development of pathologies according to results of GWASs and are (ii) characterized in detail in terms of their influence on an interaction with a TF and on the expression of nearby or distant genes. Next, the review describes the application of functional genomics methods to interpretation of data from GWASs and to the search for new regulatory variants without GWASs. In the course of the presentation, strengths and weaknesses of these approaches are shown, as is the importance of comprehensive use of GWASs and functional genomics methods to reveal the role of SNPs in molecular aberrations underlying the development of a pathology.

## Functional interpretation of data from GWASs at the level of individual rSNPs

A wide range of experimental methods are utilized to functionally study individual possible rSNPs. At initial stages, classical methods are usually used: analysis of DNA-probe retardation in a gel by nuclear extract proteins (electrophoretic mobility shift assay, EMSA) and a reporter assay (analysis of reporter gene expression under the control of allelic variants of the SNP region), which allow a researcher to detect an influence of an SNP on the binding of some TF or on reporter gene expression, respectively (Antontseva et al., 2015; Fang et al., 2017). To identify the TFs the binding sites of which are affected by nucleotide substitution, scientists perform EMSA using appropriate antibodies or purified TFs (Piedrafita et al., 1996; Knight et al., 1999; Vasiliev et al., 1999; Jiang et al., 2020), chromatin immunoprecipitation with detection of allele asymmetry in a PCR product (ChIP-AS-qPCR) (Gao et al., 2018; Choi et al., 2020; Thynn et al., 2020; Protze et al., 2022), and mass-spectrometric analysis of proteins isolated from complexes with oligonucleotides containing minor alleles (Fang et al., 2017; Liu D. et al., 2018; Choi et al., 2020).

An impact of a nucleotide substitution in the identified TFBSs on their potential target genes is confirmed by experiments with downregulation or artificial upregulation of genes of the corresponding TFs or by studying the effect of point mutations introduced into the TFBS using CRISPR/ Cas9 technology (Prestel et al., 2019; Gutierrez-Arcelus et al., 2020; Pan et al., 2020; Thynn et al., 2020; Wang Y. et al., 2020; Wang X. et al., 2021). Lately, an increasingly popular approach in this field of research on SNPs has also been the determination of allelic imbalance of the expression for SNPs located in transcribed regions of genes. Nonetheless, it is worth noting that such an SNP can be either an rSNP proper (Syddall et al., 2013; Klein et al., 2019) or a marker SNP in a linkage group with an rSNP located in a nontranscribed region (Fang et al., 2017; Li X.-X. et al., 2019; Peng et al., 2020).

Various combinations of these techniques are employed in modern research, as illustrated by the examples below.

## rs36115365

A textbook example of a well-studied rSNP is rs36115365 (G/C), which is situated at a locus associated with various types of cancer according to data from GWASs (chr5p15.33: region 2). At this locus, a correlation analysis has revealed nine SNPs (r2 > 0.60, 1000G EUR population) associated with pancreatic cancer, testicular germ cell tumor, lung cancer, and melanoma. To screen all nine SNPs for regulatory activity, EMSA and reporter assay were performed by means of eight human cell lines (Fang et al., 2017). The use of several cell lines is a common practice in such studies (Bryzgalov et al., 2013; Boldes et al., 2020) and is aimed at the highest possible coverage of events of interaction of TFs with their binding sites; the reason is substantial differences in the sets of TFs expressed in different cell types (Tobias et al., 2021). The screening analyses identified only SNP rs36115365 as potentially regulatory, and its C allele showed both much better binding to a certain protein in the EMSA and greater activation of reporter gene expression as compared to the G allele (Fang et al., 2017).

rs36115365 is located ~18 kilobase pairs (kbp) upstream of the start of the TERT gene (encoding reverse transcriptase of the telomerase complex) and approximately 5 kbp downstream of the end of the CLPTM1L gene. According to the results of ChIP-seq (chromatin immunoprecipitation followed by sequencing of DNA from the precipitates) from project ENCODE (Moore et al., 2020), this SNP’s location overlaps with many TFBSs, and this region is enriched with active chromatin histone marks, which is typical for enhancer regions. Inactivation of this region by small-interfering-RNAmediated transcriptional silencing (Malecová, Morris, 2010) results in downregulation of only the TERT gene. To identify the TF the binding site of which changes as a consequence of the substitution of G with C, the binding of proteins from nuclear extracts to oligonucleotides containing minor alleles was implemented, followed by mass-spectrometric analysis of the bound proteins. After an analysis of the obtained peptides, four TF candidates that prefer the C allele were proposed: ZNF148, VEZF1/ZNF161, ZNF281, and ZNF740. In EMSA involving specific antibodies to these TFs, only ZNF148 was confirmed, which was subsequently verified by means of the purified ZNF148 protein in an experiment. A small-interfering- RNA-mediated knockdown of ZNF148 gave a de-finitive answer because it reduced TERT expression and telomerase activity and shortened telomere length. Thus, the C allele corrects the binding site of ZNF148, enhances the expression of TERT and, as a consequence, increases the risk of carcinogenesis (Fang et al., 2017).

## rs174575

According to GWASs, rs174575 (C/G) correlates with an elevated risk of colorectal cancer (Tian et al., 2020). This SNP is located in the first intron of the delta-6-desaturase gene (FADS2) at a distance of +41.5 kbp from its transcription start site and at a distance of –178.8 kbp from the transcription start site of the gene of long noncoding RNA AP002754.2. The rs174575 region in chromatin is enriched with histone modifications characteristic of active regulatory regions (H3K4me1, H3K4me3, or H3K27ac), and judging by DNaseseq data (identification of sites of hypersensitivity to DNase I) and findings of ATAC-seq (assay for transposase-accessible chromatin), corresponds to open chromatin (ENCODE). The hypothesis of a regulatory role of rs174575 is supported by eQTL analysis data, which show an association of the G allele with overexpression of FADS2 and AP002754.2.

Computer analysis of DNA motifs using Web services Cistrome (Zheng et al., 2019) and JASPAR (Fornes et al., 2020) has revealed that in the case of rs174575, the replacement of G with C damages the binding site of TF E2F1, and the data of ChIP-seq from ENCODE, obtained on colorectal cancer LoVo cells, indicate that E2F1 is mapped to the location of this SNP. A cross-competitive EMSA has confirmed better binding of a certain nuclear extract protein to the G allele, and that this protein is E2F1 has been demonstrated by ChIPqPCR with appropriate antibodies. For instance, in cell lines with different genotypes – HCT116 (CG), SNU-C1 (CG), and HT115 (CC) – a stronger binding of E2F1 is observed in cells carrying the G allele. A study involving reporter constructs has also confirmed a higher enhancer activity of a DNA fragment containing the G allele (Tian et al., 2020).

Direct contact between the region containing rs174575 and promoters of FADS2 and AP002754.2 has been detected by the chromosome conformation capture (3C) method, and it turned out that the interaction was much more pronounced in cell lines carrying the G allele. Further experiments indicated that overexpression of AP002754.2 sharply raises the level of FADS2 expression in HCT116 and LoVo cells, and a knockdown of AP002754.2 by microRNA causes a decrease in the expression of this gene, indicating a stimulatory role of AP002754.2 in the regulation of the FADS2 gene. On the other hand, overexpression of FADS2 or AP002754.2 significantly increases the proliferation rate of HCT116 and LoVo cells, whereas a knockdown of FADS2 or AP002754.2 significantly reduces it. It has also been shown that overexpression of FADS2 and AP002754.2 accelerates tumor growth in in vivo experiments in mice. It is known that the product of the FADS2 gene is a key enzyme in the biosynthesis of polyunsaturated fatty acids, including arachidonic acid, which in turn is a precursor of prostaglandin E2 (PGE2), which promotes tumor growth and metastasis. Thus, rs174575 acts as an allele-specific enhancer that stimulates the transcription of FADS2 and AP002754.2, leading to an elevated risk of colorectal cancer in the case of the G allele (Tian et al., 2020).

## rs4903064

According to GWASs, rs4903064 (T/C) is associated with renal cell carcinoma, the most common type of kidney cancer (Scelo et al., 2017). rs4903064 is located in the third intron of the DPF3 gene, which encodes a protein of the BAF subfamily of the SWI/SNF chromatin-remodeling complex. The predominance of the C allele of rs4903064 in tumor tissue samples from patients with clear-cell renal carcinoma has been demonstrated, in contrast to both normal kidney tissues and tumor tissue from individuals with papillary renal cell carcinoma and individuals with chromophobe renal cell carcinoma. In this context, a measurement of the ratio of alleles in DPF3 pre-mRNA in patients with clear-cell renal carcinoma heterozygous for rs4903064 has confirmed a skew toward the C allele (Protze et al., 2022).

According to ATAC-seq data obtained on primary renal cancer cells, rs4903064 is located in an open chromatin region, in a putative enhancer. Given that the substitution of T with C (rs4903064) creates a potential binding site for TF HIF, a reporter analysis has been performed on HeLa and MCF-7 cell lines, showing that an increase in reporter gene expression takes place only in the case of the C allele and only when cells are treated with a specific stabilizer of HIF: dimethyloxalylglycine. Knockouts of various isoforms of HIF have revealed that the increase in reporter activity upon stimulation with dimethyloxalylglycine depends on HIF-1α. By means of ChIP-qPCR in primary renal tubular cells with different genotypes of rs4903064 (TT, CT, and CC), enhanced binding of HIF-1α and HIF-1β to risk allele C has been confirmed. To elucidate the role of overexpression of DPF3 in the development of clear-cell renal carcinoma, a knockout of DPF3 has been performed in cells of proximal renal tubules using CRISPR/Cas9 technology. It was demonstrated in that report that cells with defective expression of DPF3 grow more slowly than control clones of the corresponding cells, suggesting that increased expression of DPF3 in proximal tubule cells stimulates proliferation (Protze et al., 2022).

## rs17114036

rs17114036 (T/C) is associated with coronary heart disease and ischemic stroke according to GWASs (Dichgans et al., 2014). This SNP is located in the 5th intron of the PLPP3 gene, encoding phospholipid phosphatase 3, which inhibits inflammation of endothelium and contributes to the integrity of its monolayer (Panchatcharam et al., 2014; Wu et al., 2015). Experiments with ATAC-seq and ChIP-seq (H3K27ac and H3K4me2) performed on human aortic endothelial cells (HAECs) have identified the region containing rs17114036 as a potential enhancer. The enhancer activity of this region was confirmed by reporter analysis, whereas protective allele C (as compared to the T allele) significantly increased the activity of luciferase upon transfection of vector constructs into HAECs. A deletion of a 66-bp region containing rs17114036 by means of CRISPR/Cas9 significantly reduced the expression of PLPP3 as compared to the unedited genome and enhanced the permeability of the monolayer of edited HAECs. As a result of modeling hemodynamic processes, an increase in the activity of the studied enhancer (containing the C allele) in HAECs was shown with an 18-hour “atheroprotective” flow as compared to an “atherogenic” flow, while no effect of the T allele was found. It turned out that the substitution of the T nucleotide with C creates a binding site (CACC) for the KLF2 protein, as confirmed by ChIP-AS-qPCR analysis in HAECs heterozygous for rs17114036. Cotransfection experiments with a plasmid causing overexpression of KLF2 also showed higher luciferase activity in the case of the tested enhancer carrying the C allele (Krause et al., 2018).

## rs4407214

rs4407214 (T/G) is associated with estrogen receptor-negative breast cancer. In the HMEC cell line, an analysis of ChIP-seq data from the ENCODE project regarding locations of marks of active chromatin (H3K4m1, H3K4m2, H3K4m3, H3K9ac, H3K27ac, H3K36m3, H3K79m2, H4K20m1, EZH2, and H2AZ) has helped to find in the rs4407214 region a regulatory locus in intron 1 of the WDR43 gene (a protein-coding gene associated with rRNA processing and ribosomal biogenesis). By EMSA, the researchers showed ASB of nuclear proteins from MCF10A and CAL-51 cells to DNA probes mimicking the region of this SNP’s location in the genome. Reporter gene expression under the control of the identified regulatory region on the same cell lines was also found to be allele-dependent (Couch et al., 2016; Fachal et al., 2020).

Bioinformatic analysis was then performed using the JASPAR database and available ChIP-seq data for this region from the ENCODE project to identify the TFs the binding sites of which are altered by the G-to-T substitution (rs4407214). As a result, USF1 was identified as such a TF. A competitive EMSA with nuclear proteins isolated from CAL-51 and MCF10A cells confirmed the ASB of USF1 in the case of the G allele. It was also demonstrated that CRISPR/Cas9- mediated removal of the presumed regulatory region containing rs4407214 results in underexpression of PLB1 (phospholipase B1), which is located at a distance of ~400 kbp from WDR43.

## Mass interpretation of GWAS data by functional genomics methods

Because results of GWASs tend to associate a trait (disease) with multiple loci (Goldstein, 2009; Boyle et al., 2017), most of which in turn contain many often coinherited SNPs (Tak, Farnham, 2015; Schaid et al., 2018), it is very difficult to choose among them the potential rSNPs involved in a disease’s pathogenesis. To date, several effective experimental solutions to this problem have been developed. First of all, these are scaled up versions of the approaches utilized to investigate individual rSNPs: a reporter assay (MPRA) and methods for studying protein–nucleic acid interactions (Reel-seq, SNPseq, and SNP-SELEX) (Zhao et al., 2020; Lu et al., 2021; Yan et al., 2021).

In particular, MPRA has been successfully used to find the rSNPs that play a key role in the genetic predisposition to lupus erythematosus (Lu et al., 2021). According to GWAS findings, 3,073 SNPs in 91 loci are associated with this disease. Those researchers constructed a barcoded library containing 12,396 170-bp oligonucleotides containing in the middle all known variants of these 3,073 SNPs; these oligos were inserted upstream of a minimal promoter placed before the eGFP gene. An influence of a minor allele on enhancer activity of inserts for 51 SNPs from 27 loci was demonstrated in the GM12878 cell line. The small number of identified rSNPs can most likely be explained by transfection of only one cell line; if several cell lines had been tested, the number of rSNPs would have been much larger due to expansion of the set of TFs involved in the study. Similarly, in MPRA, 30 out of 832 SNPs (associated with melanoma risk according to GWASs) showed a significant difference in the impact of minor alleles on reporter gene expression in the UACC903 melanoma cell line (Choi et al., 2020). For one of these 30 SNPs (rs398206), which is located in intron 1 of the MX2 gene, the difference was the largest. It turned out that risk allele A of rs398206 significantly enhances the binding of TF YY1 in vitro (in EMSA) and in vivo (according to ChIP-AS-qPCR), thereby upregulating MX2 and thus contributing to the initiation of melanoma (Choi et al., 2020). Other examples can be found in refs (Ulirsch et al., 2016; Liu S. et al., 2017; Kalita et al., 2018; Klein et al., 2019).

By methodically similar Reel-seq and SNP-seq, which are based on a comparative analysis of the binding of a TF to oligonucleotides containing minor alleles, 521 potential rSNPs have been selected out of 4,316 SNPs correlating with breast cancer according to GWASs (Zhao et al., 2020) as well as 403 possible rSNPs out of 903 SNPs associated with prostate cancer (Zhang et al., 2018). The largest study involving the approach from this research group was published in 2021 (Yan et al., 2021). The approach was named SNP-SELEX. To implement it, those authors used a library of 383,544 40-bp oligonucleotides containing in the middle all possible alleles of 95,886 SNPs. SNPs were chosen based on either their association with type 2 diabetes mellitus in GWASs or localization within a 500-kbp window containing a variant associated with this pathology. By means of 270 recombinant TFs, those authors conducted a multiplex analysis of their binding to the oligonucleotides and identified 11,079 SNPs having an appreciable allele effect on binding to at least one TF.

For mass interpretation of the results of GWASs, functional genomics data from available databases are also widely used, such as data on genome-wide profiles (ChIP-seq) of TFs’ binding (Li S. et al., 2020) and of histone modifications (Jones et al., 2020), on open-chromatin genome-wide profiles (ATACseq) (Corces et al., 2020), on three-dimensional chromatin contacts (Corces et al., 2020), and on locations of enhancer and superenhancer regions (Gong et al., 2018; Sun W. et al., 2018; Nasser et al., 2021) as well as data from eQTL analysis (Gamazon et al., 2018; Zheng et al., 2019; Barbeira et al., 2021).

For example, 8,005 SNPs – either directly associated by GWASs with major depressive disorder or present in the same linkage group – have been mapped to ChIP-seq peaks obtained from a brain tissue or cells of neuronal origin by means of antibodies to 34 various TFs (Li S. et al., 2020). After that, a search was performed for binding sites of the corresponding TFs in the regions of the SNPs using a database containing 7,699 position weight matrices (Whitington et al., 2016), and it was revealed that 34 SNPs disrupt the binding sites of 15 TFs. A reporter assay confirmed the effect of an allele on gene expression for 29 SNPs. One of them, rs3101339, proved to be located at a potential binding site of TF REST in the promoter region of the NEGR1 gene, whereas the substitution of A with C considerably damaged the structure of this site, as evidenced by a decrease in reporter gene expression under the control of an insert carrying the C allele. The influence of rs3101339 on NEGR1 gene expression in vivo was confirmed by elimination of an appropriate DNA fragment via CRISPRCas9 genomic editing. Since the product of NEGR1 plays an important part in the maintenance of required density of dendritic spines, its underexpression when A is replaced by C may substantially contribute to the development of a depressive state (Li S. et al., 2020).

Data on genome-wide profiles of active-chromatin histone marks are also turning out to be very informative for the functional interpretation of GWAS results. For example, to analyze many SNPs correlating with epithelial ovarian cancer, they have been mapped in the region of ChIP-seq peaks for H3K27Ac; these peaks were obtained by the researchers in a study on 26 tissue samples of this type of cancer (Jones et al., 2020). Then, using the motifbreakR tool (Coetzee et al., 2015), among the mapped SNPs, 469 SNPs were selected in which the nucleotide substitution substantially altered a binding site of some TF. The most frequent was the change in the sequence of the binding site of TF REST, for which there are data on its tumor-suppressive and oncogenic functions (Jones et al., 2020). Besides, the use of ChIP-seq datasets on various histone modifications from relevant databases in combination with data and tools from a database of potential regulatory variants (rVarBase) (Guo et al., 2016) has made it possible to detect in superenhancers 286 and 366 possible rSNPs associated with type 2 diabetes mellitus (Sun W. et al., 2018) and coronary heart disease (Gong et al., 2018), respectively, that alter the predicted TFBSs.

## GWAS-unrelated function-based approaches to identifying potential rSNPs

Modern massive function-based approaches to the identification of potential rSNPs are mainly based on the registration of an effect of a nucleotide substitution on some molecular phenotype. This may be (i) determination (in transcriptomes) of a difference in the expression level of individual genes among homozygotes and heterozygotes for different alleles of each SNP (eQTL analysis), (ii) identification of SNPs showing asymmetry of enrichment within transcriptome data (RNAseq: ASE events) or within epigenomic data (DNase-seq, ChIP-seq, and ATAC-seq: ASB events), or (iii) determination of an influence of an allele on reporter gene expression by MPRA.

## eQTL analysis

The term “eQTL” either means that there is a correlation between a variant (eVariant) and the expression level of a certain gene(s) (eGene[s]) (GTEx Consortium, 2017, 2020) or refers directly to an SNP the alleles of which show such a correlation; the term is used much more frequently in the latter sense (Fairfax et al., 2014; Fan et al., 2020; Jiang et al., 2020; Werling et al., 2020). Transcriptomic data obtained using either microarrays (Fairfax et al., 2014; Westra, Franke, 2014) or RNA-seq (GTEx Consortium, 2020) are suitable for finding eQTLs. These data are quite sufficient for the detection of eQTLs located in transcribed regions (Göring et al., 2007), whereas the identification of their entire set also requires genome sequencing data (GTEx Consortium, 2020; Werling et al., 2020). In contrast to GWASs, which require biological samples from many thousands of individuals (Tam et al., 2019), several hundred participants are sufficient for eQTL analysis (Westra et al., 2013; Fairfax et al., 2014; GTEx Consortium, 2020). Nonetheless, just as in GWASs, in eQTL analyses, the problem of distinguishing an SNP that is indeed relevant to the formation of a trait – among marker variants detected through linkage disequilibrium – is still relevant (Zou et al., 2019; Umans et al., 2021).

The largest-scale project on obtaining transcriptomic data and identifying eQTLs is international consortium GTEx, within which RNA-seq data on 15,201 postmortem samples of 49 tissues collected from 838 donors have been collected, allowing to identify 4,278,636 eQTLs associated with changes in the expression of 18,262 and 5,006 genes encoding proteins and long intergenic noncoding RNAs, respectively (GTEx Consortium, 2020).

There are other datasets of eQTLs, including those obtained not on postmortem but on biopsy materials (Fairfax et al., 2014; Stolze et al., 2020). For example, in a study by Stolze et al., in an analysis of transcriptomes (RNA-seq) of the aortic endothelium of 157 donors, the investigators identified thousands of eQTLs not registered in the GTEx Consortium data (Stolze et al., 2020). Fairfax et al. have employed CD14+ monocytes (derived from healthy individuals) treated in vitro with either interferon gamma (for 24 h, 367 individuals) or bacterial cell wall lipopolysaccharide (LPS) (2 and 24 h, 261 and 322 individuals, respectively). CD14+ monocytes from 414 people served as controls there. Through microarray RNA profiling and genotyping, 609,704 SNPs (minor allele frequency > 0.04) were examined and 21,516 eQTLs were detected, 24.6 % of which manifested themselves in control cells, 21.6 % of which manifested themselves after 2 h of treatment with LPS, and 25.4 and 28.3 % after 24 h treatment with LPS and IFN-γ, respectively. The results of this work point to an important role of genomic variants in the nature of transcriptomic response to a drug (Fairfax et al., 2014). In conclusion, it should be noted that any dataset of transcriptomic data obtained from hundreds or more individuals can be used to search for eQTLs, as, for example, has been done by us (Korbolina et al., 2021) with the help of data from RNAseq analysis of postmortem brain samples from 96 individuals (Ramaker et al., 2017).

At present, quantitative expression trait loci analysis is mainly utilized to identify groups of genes participating in trait formation (Hormozdiari et al., 2016; Morrow et al., 2018; Gamazon et al., 2019; Ratnapriya et al., 2019; Jaffe et al., 2020). Additionally, its results are often used to prioritize GWAS-identified SNPs for their subsequent rigorous experimental investigation. An example is rs13239597, which is located in the TNPO3 gene promoter and associated with lupus erythematosus and multiple sclerosis according to GWASs. eQTL analysis of transcriptomes of lymphoblastoid cell lines derived from 373 individuals has not revealed any impact of rs13239597 on TNPO3 expression but uncovered a significant association of the A allele of this SNP with overexpression of the IRF5 gene [which is located 118 kbp away (Thynn et al., 2020)], in agreement with findings of the GTEx Consortium (GTEx Consortium, 2017). Next, an analysis of available Hi-C data showed that IRF5 is one of 12 genes that are in direct contact with the rs13239597 region. A computational analysis of motifs that potentially change affinity for a TF as a result of a nucleotide substitution (Coetzee et al., 2015) has revealed four such TFs: EVI1, ERF, GATA1, and TAL1. By ChIP-AS-qPCR, it has been demonstrated that EVI1 binds much better to the rs13239597 region in the case of the A allele as compared to the C allele (Thynn et al., 2020). Similar examples can also be found in refs (Roca-Ayats et al., 2019; Jiang et al., 2020; Tian et al., 2020).

## A large-scale search for ASE and ASB events

The development of next-generation-sequencing-based methods of transcriptomic analysis (RNA-seq) and epigenomic analysis (ChIP-seq, DNase-seq, and ATAC-seq) has opened up a unique opportunity for quantifying a difference in the enrichment of two alleles (an allele imbalance) of each heterozygous polymorphic site of a diploid organism within the respective dataset (Maurano et al., 2015; Cavalli et al., 2016a, b; Castel et al., 2020; Fan et al., 2020; Xu et al., 2020; Korbolina et al., 2021). An important feature of these approaches to the identification of potential rSNPs – in contrast to eQTL analysis (and even more so in contrast to GWASs investigating SNPs of many individuals in various genomic contexts and living conditions) – is that allele-asymmetric events are recorded for each individual and the backgrounds are identical. This arrangement enables researchers to obtain reliable data when studying a very small number of individuals, down to one (Harvey et al., 2015). An increase in sample size is required only for involving in the analysis a larger number of SNPs that are in a heterozygous state. For instance, calculations show that data from 20 individuals theoretically allow to determine ASE or ASB events for 65–70 % of SNPs that have a population frequency of ≥5 % (Cavalli et al., 2016a).

For this reason, possibilities of pharmacogenetic and pharmacogenomic projects become much more abundant. The most striking example of such a project is a simultaneous analysis of allele-specific effects of 50 substances (steroid and peptide hormones, nutrients, commonly used drugs, and a number of environmental pollutants) in primary cultures of five cell types (LCLs, PBMCs, HUVECs, SMCs, and melanocytes), each of which is represented by cell samples from three individuals (Moyerbrailean et al., 2016). An analysis of the resultant transcriptomic data helped to identify more than 300 SNPs, an imbalance in the enrichment of the alleles of which within transcriptomes emerged or increased significantly in response to treatment with one or another drug. Via the same approach, inducer (LPS of the bacterial cell wall)-dependent ASE events have been identified in 19 immune response genes by an analysis of transcriptomes of blood mononuclear cells from eight individuals (Edsgärd et al., 2016) as well as 561 ASE events responsive to treatment of CD4+ T cells (from 24 genotyped individuals) with immobilized anti-CD3/CD28 antibodies (Gutierrez-Arcelus et al., 2020). These results open up a new – unrelated to any a priori hypothesis – way to elucidate mechanisms of individual sensitivity to drugs

The largest dataset of ASE events at present, containing 431 million such events, is derived from data on RNA-seq and whole-genome sequencing from the GTEx Consortium (GTEx Consortium, 2020) and is published in a paper by Castel et al. (Castel et al., 2020). This dataset can serve as a source for mass discovery of rSNPs, for example, in a comparison with data from GWASs or from eQTL analysis. It is worth noting that in the absence of whole-genome sequencing data, relevant information can be acquired by more sophisticated methods of bioinformatic search for allele-specific events directly in RNA-seq data (Harvey et al., 2015; Moyerbrailean et al., 2016; Fan et al., 2020; Korbolina et al., 2021).

ChIP-seq experiments based on antibodies to various TFs make it possible to directly register events of allele-asymmetric interaction of these proteins with their binding sites in the case of a heterozygous state of SNPs at these sites. In a pioneering work in the laboratory of Claus Wadelius, they analyzed all the then-available data from the ENCODE project on binding profiles of TFs in cell lines GM12878 (B cells), H1-hESC, K562, and SK-N-SH, thereby revealing 9,962 SNPs featuring an allelic imbalance in the binding of a TF (ASB) (Cavalli et al., 2016b). By the same approach, 3,713 SNPs have been found showing an allelic imbalance in the binding of TFs in HepG2 and HeLa-S3 cells; testing 39 of them in a luciferase reporter system has confirmed the effect of an allele on reporter gene expression for 27 SNPs (Cavalli et al., 2016a). A detailed analysis of one of them, rs953413, indicates that the A allele disrupts the binding site of TF FOXA, resulting in reduced binding of not only this TF but also of TF HNF4α cooperatively interacting with it, thereby ultimately leading to underexpression of ELOVL2 and possibly serving as a factor in the pathogenesis of non-alcoholic fatty liver disease (Pan et al., 2020).

Because allele-asymmetric alterations in profiles of histone modifications and of open chromatin can reflect SNP-induced changes in the binding of TFs (Kar et al., 2014; Hatayama, Aruga, 2018; Huang et al., 2018; Yi et al., 2020), these data are also widely utilized to find ASB events. For instance, Maurano et al. (Maurano et al., 2015) have examined 493 openchromatin profiles (DNase-seq) obtained in various cell lines, where 64,599 SNPs with ASB have been found. Their bioinformatic analysis using position weight matrices for 2,203 motifs of TFBSs from different sources indicates that most of the identified SNPs can affect the binding of TFs and hence the accessibility of the respective DNA regions to DNase I (Maurano et al., 2015). A newer technique for detecting open chromatin, ATAC-seq, is based on the ability of a hyperactive mutant of Tn5 transposase to detect open DNA regions in chromatin (Marinov, Shipony, 2021) and is also used to search for ASB events. In particular, it has been employed to identify 53 rSNPs in breast cancer MCF-7 cells and 125 rSNPs in a line of human mesenchymal stem cells (MSCs); in total, 30 % of the rSNPs found in MCF-7 cells and 43 % of those found in MSCs have been identified as eQTLs in GTEx data, indicating their influence on gene expression (Xu et al., 2020). Examples of use of data from ChIP-seq (involving antibodies to histone marks) for registering ASB events can be found in refs (Sun J. et al., 2016; D’Oliveira Albanus et al., 2021; Li M. et al., 2021).

The combination of searches for ASE events and ASB events can be considered the most productive approach to identifying rSNPs. For example, in our work (Korbolina et al., 2018), at first, ASB events were identified in ChIP-seq data from the ENCODE project for histone modifications H3K27ac, H3K4me1, H3K4me2, H3K4me3, and H3K27me3 as well as for 456 TFs and their associated proteins in human cell lines K562, MCF-7, and HCT-116. Then, by means of RNA-seq data obtained from the same cell lines, SNPs (rSNPs) that are associated with changes in gene expression levels were identified. According to GWASs, out of 1,633 rSNPs found in this way, 27 have shown associations with cancers (Korbolina et al., 2018), and 14, with cognitive disorders (Bryzgalov et al., 2018). Another 30 rSNPs have been implicated in colorectal cancer with the help of data from the International Cancer Genome Consortium (ICGC) (Seshagiri et al., 2012). Genotyping of patients with colorectal cancer and healthy individuals for six of these SNPs has revealed an association of rs590352, rs4796672, and rs2072580 with this disease (Leberfarb et al., 2020). A correlation with breast cancer has been found for rs2072580 (Degtyareva et al., 2020). Later, the same approach has allowed to identify 14,266 rSNPs during the processing of data obtained in a study by Reyes-Palomares et al. (Reyes- Palomares et al., 2020) on H3K4me3 histone marker profiling (ChIP-seq) and RNA-seq data on pulmonary-artery epithelial samples from 19 individuals (Korbolina et al., 2021).

## MPRA

Research into the effect of polymorphic-site alleles on reporter gene expression via simultaneous transfection of hundreds and thousands of barcoded plasmid constructs into eukaryotic cells with subsequent transcriptome sequencing is also an informative approach to finding rSNPs (Vockley et al., 2015; Tewhey et al., 2016; Movva et al., 2019). The largest-scale study based on this approach has been conducted in the laboratory of Bas van Steensel (van Arensbergen et al., 2019). Using a promoterless plasmid and fragmented genomes (fragment length 150–500 bp) of four individuals belonging to different ethnic groups, two barcoded libraries were constructed for each individual, where inserts were expected to play the role of a promoter. At the same time, on the basis of data on transcription initiation in enhancer regions (Natoli, Andrau, 2012; van Arensbergen et al., 2017), those authors expected to detect not only promoters but also enhancers. The use of DNA from humans of genetically distant ethnic groups allowed those investigators to hope for an analysis of the largest possible number of polymorphic sites that are homozygous for different alleles in at least two of those people.

After transfection of K562 and HepG2 cells with the resulting libraries, 19 and 14 thousand potential rSNPs, respectively, were found, most of which did not overlap, once again indicating tissue specificity of the supragenomic (protein) regulatory machine. The identified SNPs showed significant enrichment within regulatory regions of the genome. In this case, the enrichment (approximately 15-fold) was three times higher in promoter regions than in enhancer regions (approximately 5-fold); this outcome is obviously due to the design of the reporter constructs. For several rSNPs, by mass-spectrometric analysis of proteins interacting with oligonucleotides containing minor alleles, those authors were able to identify TFs the binding sites of which are altered by a nucleotide substitution. In particular, the A allele of rs623853 was found to disrupt the binding of TFs of the ELF family, whereas the C allele of rs554591 weakens the binding of ZNF787 while enhancing the binding of KLF and SP (van Arensbergen et al., 2019).

## Conclusion

**Fig. 2.**
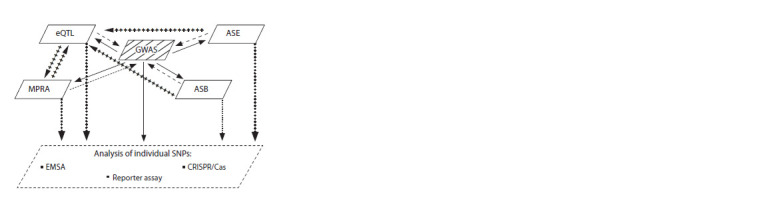
The scheme of an integrative approach to the search for SNPs functionally important for the development of a trait (pathology) on the basis of two opposite principles: from an association with a trait to its function (solid arrows) and vice versa, from a function to a trait (dashed arrows). Cross-containing arrows show ways to increase the evidence base for functional significance of sets of SNPs, and dotted arrows represent ways to study individual SNPs in detail.

Programs – of coordinated switching on, switching off, and changes of the expression of various genes – that underlie (1) ontogenesis events, (2) the existence of many types of differentiated cells, and (3) the abilities of cells to respond to various factors of the external and internal environment are implemented by the regulatory part of the genome of multicellular organisms. The information encoded in the regulatory regions is converted into a desired pattern of gene expression primarily via the binding of TFs to specific sequences in the regulatory regions (e. g., promoters, enhancers, and silencers) (Lan et al., 2012; Merkulova et al., 2013; Dubois-Chevalier et al., 2018; Chen, Pugh, 2021; Tobias et al., 2021). According to present-day concepts, the SNPs located in regulatory regions of genes, affecting binding sites of TFs, and changing the level of gene expression play a central part in the variation of phenotypic traits, including predisposition/resistance to multifactorial diseases (Maurano et al., 2015; Deplancke et al., 2016; Carrasco Pro et al., 2020). In this regard, there is a strong interest both in functional interpretation of SNPs having an association with various diseases (primarily according to GWAS data) and in the development of massive function-based approaches to the discovery of rSNPs. Interpretation of data from GWASs is carried out either at the level of individual SNPs or for all SNPs collectively by a variety of functional genomics techniques (Fig. 2). Meanwhile, the same methods of functional genomics are used for personal searches for rSNPs, but at the same time, it is necessary to solve the inverse problem: determining a relation between the found rSNPs and a trait (disease). The most popular solution to this problem is to compare the obtained data with available information from GWASs (see Fig. 2). On the other hand, in this way, usually only 1.5–3.0 % of found rSNPs are implicated in various traits (Cavalli et al., 2016b, 2019; Korbolina et al., 2021). In this regard, it seems very promising to take advantage of results of eQTL analysis, enabling an investigator to determine an influence of many specific rSNPs on the expression of fairly large groups of genes, the subsequent analysis of which by modern functional annotation tools (Gene Ontology, Kyoto Encyclopedia of Genes and Genomes, and others) allows to get an idea about a possible affected trait (Korbolina et al., 2021).

To sum up, there are two approaches – based on opposite principles – to finding SNPs that are important for the development of a trait (pathology): on the one hand, starting from data on an association of an SNP with some trait, and on the other hand, starting from determination of allele-specific changes at the molecular level (in the transcriptome or regulome). It can be concluded that comprehensive use of the two approaches appreciably enriches our knowledge about the participation of genetic determinants in molecular mechanisms of trait formation, including predisposition to multifactorial diseases.

## Conflict of interest

The authors declare no conflict of interest.
